# Fahr's Syndrome in a Young Adult Male: A Case of Seizure and Cognitive Decline Secondary to Hypoparathyroidism

**DOI:** 10.1002/ccr3.72206

**Published:** 2026-03-11

**Authors:** Sarah Nisar, Hamza Mushtaq, Muhammad Haris Khan, Fatima Sajjad, Abdullah Afridi, Zaryab Bacha, Bibi Hafza, Kamil Ahmad Kamil

**Affiliations:** ^1^ Khyber Teaching Hospital Peshawar Pakistan; ^2^ Sebring Advent Health Sebring Florida USA; ^3^ Saidu Medical College Swat Pakistan; ^4^ Khyber Medical College Peshawar Pakistan; ^5^ Internal Medicine Department Mirwais Regional Hospital Kandahar Afghanistan

**Keywords:** CT imaging, Fahr's syndrome, hypoparathyroidism, intracranial calcification, seizures

## Abstract

Fahr's syndrome is a rare neurological condition characterized by bilateral intracranial calcifications secondary to metabolic or endocrine abnormalities, most commonly hypoparathyroidism. This condition is distinct from Fahr's disease (primary familial brain calcification), which is genetically inherited. A 30‐year‐old South Asian Pakistani man presented with sudden‐onset generalized tonic–clonic seizures and progressive cognitive decline. Neuroimaging revealed bilateral, symmetrical calcifications in the basal ganglia, thalami, cerebellar dentate nuclei, periventricular regions, and centrum semiovale. Laboratory evaluation revealed severe hypocalcemia, hypomagnesemia, and markedly reduced parathyroid hormone levels, consistent with hypoparathyroidism. The patient was treated with calcium supplementation, calcitriol, vitamin D, magnesium replacement, and antiepileptic therapy, resulting in clinical stabilization of the patient. Fahr's syndrome should be considered in young patients presenting with seizures and cognitive impairment when characteristic intracranial calcifications are identified. Early recognition and correction of metabolic abnormalities can improve the outcomes.

**Key Clinical Message**

Fahr's syndrome should be considered in patients presenting with seizures or neuropsychiatric symptoms and bilateral basal ganglia calcifications. Prompt correction of hypocalcemia, hypoparathyroidism, and associated electrolyte abnormalities may prevent disease progression in these patients.

**FIGURE 1:**
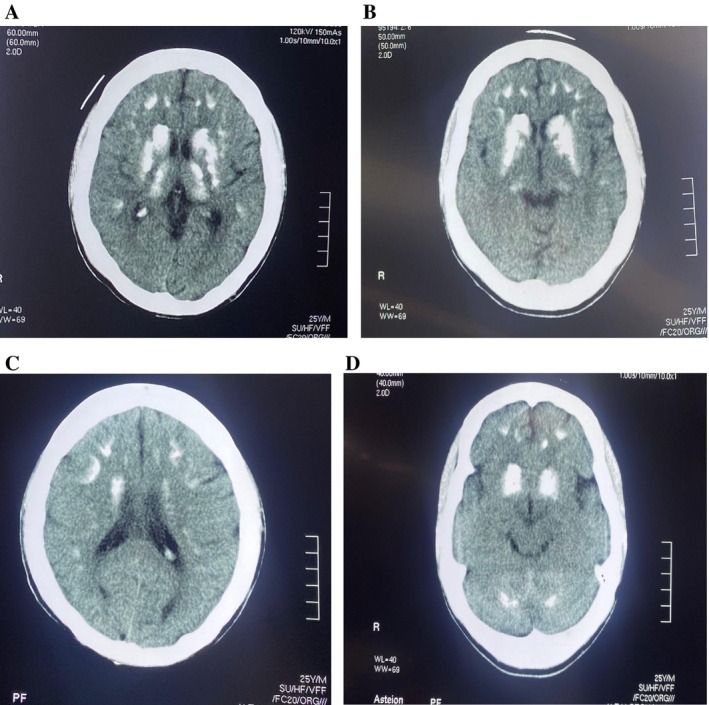
(A) CT brain revealed bilateral calcification in Basal ganglia, thalami, and cerebellar dentate nuclei. (B) CT brain revealed bilateral calcification in Basal ganglia and the Thalamus. (C) CT brain revealed bilateral calcification in Subcortical white matter, basal ganglia and periventricular region. (D) CT brain revealed bilateral calcification in Basal ganglia particularly the globus pallidus and cerebellar dentate nuclei.

## 1 Introduction

Fahr's syndrome is a rare sporadic, autosomal dominant or autosomal recessive inherited neurodegenerative disorder characterized by bilateral calcification, particularly involving the intracranial region [1, 2]. A German Neurologist, Karl Theodor Fahr first illustrated Fahr's syndrome in 1930 [3]. This condition involves the abnormal accumulation of calcium in brain regions that control motor functions, including the thalamus, basal ganglia, cerebral cortex, dentate nucleus, hippocampus, subcortical white matter, and cerebellum [4].

Three primary genetic loci have been implicated in this syndrome: chromosome 8, chromosome 2, and, most commonly, the 4q locus (IBGC1). Only one of these three loci is associated with Fahr Syndrome [5, 6]. It is typically associated with endocrine metabolic abnormalities, such as hypoparathyroidism, pseudohypoparathyroidism, idiopathic hypoparathyroidism, and secondary hypoparathyroidism [1]. Fahr's syndrome is distinct from Fahr's disease, also known as primary familial brain calcification, which is genetically inherited. Unlike Fahr's disease, Fahr's syndrome may be partially reversible with timely correction of the underlying metabolic abnormalities [6]. It is a male‐dominant disease with a reported male‐to‐female ratio of approximately 2:1, and its overall prevalence is estimated to be less than 1 in 1,000,000 [7]. The symptoms involve disorders of motor functions, including parkinsonism, chorea, athetosis, speech disorders, and cerebellar symptoms, as well as neuropsychiatric symptoms, mainly anxiety, mania, and hallucinations [8, 9].

We report a case of a 30‐year‐old male who presented with a generalized tonic clonic seizure. Further investigations revealed bilateral symmetrical dense calcification in the basal ganglia, thalami, and periventricular areas on neuroimaging, with associated hypocalcemia and low parathyroid hormone levels.

## 2 Case History

A 30‐year‐old man with no smoking history and prior comorbidities presented to the emergency department with sudden onset of generalized tonic clonic seizures lasting approximately 4 min. The episode was followed by rigidity, frothing of the mouth, and clumsy movements. The patient regained consciousness within 10 min. Notably, the event was not accompanied by tongue biting, urinary incontinence or fecal incontinence.

The patient reported a history of recurrent seizures beginning 10–12 years earlier, for which he had started antiepileptic therapy with sodium valproate (Epival) at a dose of 500 mg. However, no records regarding the specific diagnosis, treatment regimen, or response to therapy were available for review. The patient reported a gradual and progressive decline in cognitive function over approximately 6–8 years before the current presentation. This initially manifested as short‐term memory impairment, difficulty retaining new information, and a reduced attention span, which progressively interfered with daily activities. Over time, he also experienced slowed information processing and decreased executive functioning, as reported by family members. There was no history of acute confusional episodes, personality changes, or psychotic symptoms in the patient. Formal neuropsychological testing could not be performed due to resource limitations; however, the clinical course was consistent with slowly progressive cognitive impairment, likely secondary to chronic metabolic derangements associated with hypoparathyroidism and intracranial calcification. There was no known family history of epilepsy, neurodegenerative disorders, or endocrine abnormalities, and no relatives had experienced similar symptoms.

### 2.1 Differential Diagnosis, Investigations and Treatment

On clinical examination, the patient was afebrile, with a blood pressure of 110/70 mmHg, pulse rate of 86 beats per minute, and oxygen saturation of 96% on room air. The random blood glucose level was 146 mg/dL. The Glasgow Coma Scale (GCS) score was 15/15, and the patient was drowsy but arousable. Deep tendon reflexes were reduced. No focal neurological deficits or indications of cerebellar dysfunction were noted.

Laboratory investigations revealed significant hypocalcemia, with a serum calcium level of 3.4 mg/dL (Normal Range: 8.0–10.0 mg/dL), accompanied by severe hypomagnesemia with a serum magnesium level of 0.2 mmol/L (normal range: 0.66–1.03 mmol/L). The serum phosphate level was within normal limits at 0.98 mmol/L (Normal Range: 0.8–1.4 mmol/L). Hormonal evaluation showed a significantly reduced level of intact parathyroid hormone (PTH), measured at 1.1 pg/mL (reference range: 10.0–69.0 pg/mL), despite normal circulating vitamin D levels. Other biochemical parameters, including serum electrolytes, liver function tests, renal profile, and lipid panel, were within their respective normal ranges.

Chvostek's and Trousseau's signs were positive. Non‐contrast axial computed tomography (CT) of the brain revealed bilateral symmetrical dense calcifications in the basal ganglia, thalami, periventricular areas, centrum semiovale, and cerebellar dentate nuclei, consistent with Fahr's syndrome (Figure 1A–D). There was no evidence of hemorrhage or infarction, and no parenchymal hypodensity was observed.

Electrocardiography (ECG) revealed a sinus rhythm with a corrected QT interval (QTc) of 458 ms. Electroencephalography (EEG) was performed after stabilization of the acute seizure episode and correction of the metabolic abnormalities. The EEG showed no epileptiform discharges or focal slowing, and the background activity was within normal limits. These findings suggested the absence of an underlying primary epileptic focus, supporting the interpretation that the seizure activity was provoked by severe metabolic disturbances, particularly hypocalcemia and hypomagnesemia, rather than a primary epilepsy syndrome. Genetic evaluation test was not performed due to limited financial resources. These findings led to the diagnosis of Fahr's syndrome.

The patient was treated with oral calcium carbonate (1000 mg daily), calcitriol (0.5 μg daily), vitamin D3, and levetiracetam. Given the presence of severe hypomagnesemia (serum magnesium level: 0.2 mmol/L), magnesium replacement therapy was initiated promptly. The patient received intravenous magnesium sulfate during hospitalization, followed by oral magnesium supplementation after stabilization. Correction of serum magnesium levels was prioritized because of its role in parathyroid hormone secretion and calcium homeostasis.

## 3 Outcome and Follow‐Up

Subsequent laboratory monitoring demonstrated gradual normalization of magnesium levels, which facilitated improvement in serum calcium concentrations when combined with calcium carbonate and calcitriol therapy. He remained seizure‐free on follow‐up with stabilization of cognitive symptoms.

## 4 Discussion

Fahr's syndrome is a neurodegenerative disorder characterized by symmetrically arranged calcifications in the basal ganglia, thalamus, dentate nuclei, and other cerebral structures. Its prevalence is estimated to be less than 1/1,000,000 and its pathogenesis is closely linked to metabolic or endocrine abnormalities [10, 11]. The current case presentation, associated with a 30‐year‐old man who suddenly developed generalized tonic–clonic seizures and at the same time exhibited a pattern of progressive intellectual decline, reflects the multifactorial clinical picture and the diagnostic difficulties in the context of Fahr's syndrome.

The current case depicts the diagnostic difficulties experienced in Fahr's syndrome. The patient had no known history of smoking, comorbidities, or family history of neurological or endocrine diseases. A severe acute neurological episode was followed by constant rigidity, which was also cumulated by a longstanding history of epilepsy that was being treated using antiepileptic drugs. Although the further clinical picture included severe cognitive dysfunction with memory and attention processes, the preliminary diagnostic study of the patient was not completed. This example illustrates that neuropsychiatric symptoms, such as mood changes, concentration impairments, and psychiatric symptoms, such as hallucinations or mania, can be followed by motor abnormalities or seizures in several years, thus taking part in a delayed diagnosis process [10, 12, 13].

Hypoparathyroidism is the most common reversible metabolic precipitating factor of Fahr's syndrome, caused by chronic disturbance of calcium and phosphate balance [11, 14, 15]. Severe hypomagnesemia, as seen in this patient, can impair parathyroid hormone secretion and action, leading to functional hypoparathyroidism and exacerbating hypocalcemia and seizures. The initial laboratory results in the present case included low serum parathyroid hormone levels and hypocalcemia, which also support this diagnosis. The mildly prolonged QTc interval observed in this patient was consistent with severe hypocalcemia and underscores the potential risk of cardiac arrhythmias. Hypocalcemia is a well‐recognized cause of QTc prolongation and predisposes patients to ventricular arrhythmias, with QTc duration correlating with the degree of hypocalcemia [16, 17]. Radiographically, bilateral, symmetrical calcification of the basal ganglia is a strong clinical and diagnostic indication, especially in the presence of neuropsychiatric symptoms and seizures [18, 19]. This pattern commonly extends to the thalami, dentate nuclei, and subcortical white matter as revealed in this case.

Fahr's disease may be genetic in nature and linked to mutations in SLC20A2, exhibiting autosomal dominant inheritance. However, the lack of a familial pattern and the presence of secondary metabolic disorders in the index case can aid in the diagnosis of Fahr's syndrome [20].

Correction of the underlying metabolic disturbance was the main management goal in the current case report. Stabilization of neurocognitive functioning and termination of seizures were achieved by normalizing serum calcium and vitamin D levels with the help of calcium carbonate, calcitriol, and cholecalciferol, as well as levetiracetam [21, 22]. Biochemical monitoring is essential and should be continuous, as changes in calcium or parathyroid hormone levels can trigger the onset of neuropsychiatric worsening [14, 23]. In addition, a multidimensional treatment plan, which includes psychiatric treatment when needed, has a presumed positive effect on patient outcomes and general quality of life [12, 13, 24].

Fahr's syndrome must be considered in the differential diagnosis of patients presenting with unexplained seizures, movement disorders, or neuropsychiatric symptoms, especially when typical brain calcifications are identified on scans. Metabolic testing and secondary etiology determination at an early age will allow early intervention and potentially limit the progressive course of the disorder [12, 19, 22]. A male predominance with a male‐to‐female ratio of approximately 2:1 has been consistently reported [10, 20]. No relationship between the degree of calcification and neurological deficit has been determined [12, 13].

In discussing the course of this patient, the importance of a thorough neurologic, psychiatric, and metabolic workup in suspected instances can be underscored, which adds to a more comprehensive clinical and treatment approach to Fahr's syndrome.

## Author Contributions

**Sarah Nisar:** supervision, writing – original draft. **Hamza Mushtaq:** writing – review and editing. **Muhammad Haris Khan:** conceptualization, writing – original draft. **Fatima Sajjad:** conceptualization, project administration, writing – original draft. **Abdullah Afridi:** investigation. **Zaryab Bacha:** supervision, writing – original draft. **Bibi Hafza:** writing – review and editing. **Kamil Ahmad Kamil:** conceptualization.

## Funding

The authors have nothing to report.

## Consent

Written informed consent was obtained from the patient for publication of this case report and any accompanying images.

## Conflicts of Interest

The authors declare no conflicts of interest.

## Data Availability

The data that support the findings of this study are available on request from the corresponding author. The data are not publicly available due to privacy or ethical restrictions.
